# Beneficial Effects of Ethyl Pyruvate through Inhibiting High-Mobility Group Box 1 Expression and TLR4/NF-*κ*B Pathway after Traumatic Brain Injury in the Rat

**DOI:** 10.1155/2011/807142

**Published:** 2011-06-16

**Authors:** Xingfen Su, Handong Wang, Jinbing Zhao, Hao Pan, Lei Mao

**Affiliations:** Department of Neurosurgery, Jinling Hospital, School of Medicine, Nanjing University, 305 East Zhongshan Road, Jiangsu Province, Nanjing 210002, China

## Abstract

Ethyl pyruvate (EP) has demonstrated neuroprotective effects against acute brain injury through its anti-inflammatory action. The nuclear protein high-mobility group box 1 (HMGB1) can activate inflammatory pathways when released from dying cells. This study was designed to investigate the protective effects of EP against secondary brain injury in rats after Traumatic Brain Injury (TBI). Adult male rats were randomly divided into three groups: (1) Sham + vehicle group, (2) TBI + vehicle group, and (3) TBI + EP group (*n* = 30 per group). Right parietal cortical contusion was made by using a weight-dropping TBI method. In TBI + EP group, EP was administered intraperitoneally at a dosage of 75 mg/kg at 5 min, 1 and 6 h after TBI. Brain samples were harvested at 24 h after TBI. We found that EP treatment markedly inhibited the expressions of HMGB1 and TLR4, NF-*κ*B DNA binding activity and inflammatory mediators, such as IL-1*β*, TNF-*α* and IL-6. Also, EP treatment significantly ameliorated beam walking performance, brain edema, and cortical apoptotic cell death. These results suggest that the protective effects of EP may be mediated by the reduction of HMGB1/TLR4/NF-*κ*B-mediated inflammatory response in the injured rat brain.

## 1. Introduction

Traumatic brain injury (TBI) usually leads to devastating neurological deficits and disabilities [1]. Yet, there is current lacking of effective treatment that can improve clinical outcome. TBI has been classified into primary injury and secondary injury [2]. The primary injury is understood as the result of the mechanical forces acting on the injured brain. The secondary injury is due to the complex biochemical and pathophysiological changes that follow the primary insults [3]. Of the many pathophysiological events that may contribute to this delayed injury, posttraumatic inflammation and apoptosis have been suggested to contribute to the secondary brain injury and to result in a worsening of neurologic outcome [4, 5].

High-mobility group box 1 (HMGB1) was originally identified as a DNA-binding protein that functions as a structural cofactor critical for proper transcriptional regulation in somatic cells [6]. HMGB1 can be actively and passively released from the necrotic cells other than apoptotic cells, and then triggers inflammation [7, 8]. Recently, much evidence identifies HMGB1 as a cytokine-like mediator [9, 10]. The extracellular HMGB1 binds to the receptor for advanced glycation end products (RAGE) and other receptors, including TLR2 and TLR4 [6, 11]. And then these receptors activate a common signaling pathway that culminates in the activation of nuclear factor-kappa B (NF-*κ*B) transcription factors. As one of the most important downstream molecules in TLRs signaling pathway, NF-*κ*B is a transcriptional factor required for the gene expression of many inflammatory mediators, such as interleukin-1*β* (IL-1*β*), tumor necrosis factor-*α* (TNF-*α*), and interleukin-6 (IL-6) [12]. Our previous unpublished data suggested that HMGB1 was upregulated and relocated after TBI. HMGB1 may implicate an important role in promoting inflammation after TBI. 

Ethyl pyruvate (EP) is a stable and lipophilic ester derived from the endogenous metabolite pyruvic acid [13]. The pharmacological effects of EP include downregulation of the secretion of proinflammatory cytokines, amelioration of redox-mediated damage to cells and tissues, and inhibition of apoptosis [14]. EP protects various inflammatory tissue injuries, such as lethal sepsis and systemic inflammation [15], hemorrhagic shock [16], and stroke [17]. 

Several previous studies have demonstrated that treatment with a solution containing EP down-regulated activation of the proinflammatory transcription factor, NF-*κ*B, as well as the expression of several proinflammatory proteins, such as TNF-*α*, IL-6 [16, 18]. Recently, the protective effect of EP on spinal cord injury and brain trauma has been reported [19, 20]. Nevertheless till now, it is still unknown whether EP can influence HMGB1/TLR4/NF-*κ*B pathway and the production of inflammatory agents in the brain tissue after TBI. And EP was the first described pharmacological inhibitor for HMGB1 secretion [13]. Thus, the aim of the current study was to determine whether EP could attenuate the TBI-induced activation of HMGB1/TLR4/NF-*κ*B signaling pathway in the pericontusion area. 

## 2. Materials and Methods

### 2.1. Animal Preparation

Male *Sprague-Dawley* rats (250 to 300 g) were purchased from Animal Center of Chinese Academy of Sciences, Shanghai, China. The rats were raised on a 12-hour dark-light cycle circumstance with free access to food and water. The protocols including all surgical procedures and animal usages were approved by the Animal Care and Use Committee of Nanjing University and conformed to the Guide for the Care and Use of Laboratory Animals by the National Institutes of Health. 

### 2.2. Experimental Protocols

A minor modification of the Feeney's weight-drop model was used to induce rat model of cortical contusion trauma [21, 22]. After intraperitoneal anesthesia with pentobarbital sodium (50 mg/kg, i.p.), rats were fixed in the stereotactic apparatus. The scalp was cleaned with iodophor, and aseptic techniques were used throughout surgery. The scalp was cut open, and a 5 mm diameter bone window (the center of the bone window was 1.5 mm posterior and 2.5 mm lateral to the bregma) was drilled on the right side of the parietal skull. The dura was exposed and kept intact. Focal brain injury was induced by dropping a 40 g steel rod with a flat end from a height of 25 cm onto a piston (diameter 4 mm) resting on the dura. The piston was allowed to compress brain tissue for a maximum depth of 5 mm. Then the scalp was sutured. Sham-operated rats were anesthetized and mounted in the stereotactic apparatus, with right parietal craniotomy surgically prepared alone and without suffering the cortical contusion. During recovery from anesthesia, the rats were placed on a warm heating pad and covered with a warm towel. And then the rats were returned to their cages and the room temperature kept at 23 ± 1°C. 

Rats were randomly allocated into the following groups: (a) TBI + EP group: the rats were subjected to TBI and administered EP solution (dissolved in 0.9% saline solution, 10 mg/mL) at a dosage of 75 mg/kg via intraperitoneal injection (i.p.) 5 min, 1 hour, and 6 hours after TBI (*n* = 30 total); (b) TBI group: the rats were subjected to TBI plus administration (i.p.) equal volumes of 0.9% saline solution (7.5 mL/kg) without EP, 5 min, 1 hour, and 6 hours after TBI (*n* = 30 total); (c) Sham group: rats were subjected to the surgical procedures as the above groups except for the focal brain injury and to these rats were administered equal volumes of 0.9% saline solution (7.5 mL/kg) without EP (administered i.p., 5 min, 1 hour, and 6 hours after surgical procedure) (*n* = 30 total). The dose and time-point were chosen with minor change according to previous studies [19, 23].

**Figure 1:**
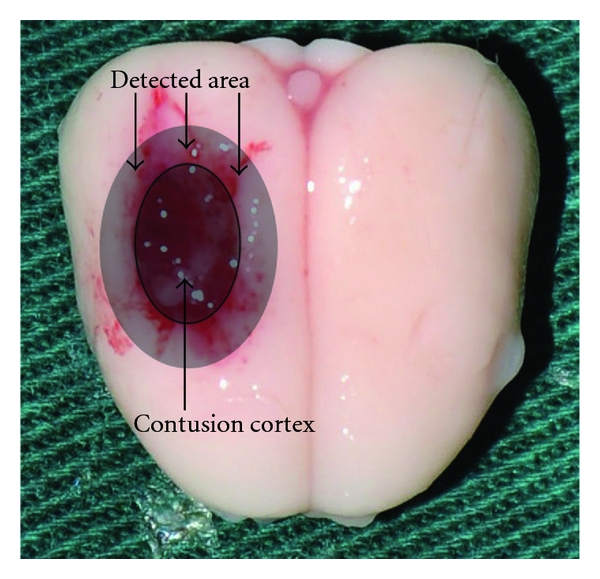
Schematic representation of the contusion cortex induced by weight-dropping trauma and the detected area surrounding the injured brain cortex.

At 24 hours after surgery, beam-walking task was conducted to test the function deficits of the rats. And then the rats were killed by an over dose of anesthesia agent. The surrounding brain tissue of the injured cortex (Figure 1) was dissected from the region that was less than 3 mm from the margin of the contusion site on ice and stored in liquid nitrogen immediately until use. Six rats in each group were sacrificed for Western bolt and enzyme-linked immunosorbent assay (ELISA), six for RT-PCR, six for electrophoretic mobility shift assay (EMSA), six for brain edema, and the others were for immunohistochemistry study. For immunohistochemistry study, the rats were transcardially perfused with 0.1 mol/L phosphate buffered saline (PBS, 4°C) and followed by 4% buffered formaldehyde; the brain was immersed in 4% buffered formaldehyde overnight and then embedded in paraffin. 

### 2.3. Beam Walking Balance Task

Beam walking balance task was used to assess locomotor function of rats after TBI as previous described [24]. The rats underwent pretraining on the wooden beam for 3 days prior to randomization. All rats had a normal score (7) before surgery. The assessment was performed at 24 hours after surgery before sacrifice. In training and testing, rats were places on one end of the 120 cm long, 2.5 cm wide, elevated (50 cm) wooden beam and forced to enter a dark goal box (25 cm wide, 25 cm long, and 25 cm high). A 60 W light source was positioned close to the starting point of the beam and provided the only illumination in the room during test sessions, and white noise (60 dB) was used to encourage locomotion. The noise was terminated after the animal traversed the beam into a dark goal box. The time to transverse the beam and enter the dark box was also recorded, and a trial was ended if the animal had not entered the goal within 90 s. Beam-walking performance was rated using a 7-point rating scale, similar to those previously published articles [24, 25]: (1) the rat is unable to place the affected hind limb on the horizontal surface of the beam; (2) it places the affected hind limb on the horizontal surface of the beam and maintains balance but is unable to traverse the beam; (3) the rat traverses the beam dragging the affected hind limb; (4) it traverses the beam and once places the affected hind limb on the horizontal surface of the beam; (5) the rat crosses the beam and places the affected hind limb on the horizontal surface of the beam to aid less than half its steps; (6) the rat uses the affected hind limb to aid more than half its steps, and (7) the rat traverses the beam with no more than 2 foot slips. The beam-walking test was performed by an independent observer, who had no prior knowledge of the experimental protocol and was unaware of the grouping. 

### 2.4. Brain Water Content

Brain edema was determined using the wet-dry method as previously described [26]. Briefly, brain tissues around the contusion cortex were rapidly harvested and immediately weighed to gain the wet weight. The samples were dried in oven at 110°C for 24 hours, and then weighed again to get the dry weight. The brain water edema was calculated by the formula: percentage of brain water = [(wet weight − dry weight)/wet weight] × 100%.

### 2.5. RT-PCR Analysis

The levels of HMGB1 and TLR4 mRNA expression were determined by RT-PCR. Total RNA was extracted with TRIzol reagent (Invitrogen Corporation, Carlsbad, CA, USA) according to the manufacturer's instructions. Total cDNA was synthesized from the isolated total RNA using a reverse transcriptional system. Briefly, 5 *μ*g of total RNA was reverse transcribed using 0.5 *μ*g oligo(dT)15 U avian myeloblastosis virus reverse transcriptase (Biouniquer Technology CO, LTD). The cDNA was then amplified by PCR. The forward and reverse primers were as follows: 5′-ATGGGCAAAGGAGATCCTA-3′ and 5′-ATTCATCATCATCATCTTCT-3′ for HMGB1 (646 bp), 5′-TTGCCTTCATTACAGGGACTT-3′ and 5′-CAGAGCGGCTACTCAGAAACT-3′ for TLR4 (179 bp), and 5′-GCCATGTACGTAGCCATCCA-3′ and 5′-GAACCGCTCATTGCCGATAG-3′ for *β*-actin (375 bp) [27, 28]. PCR amplification was performed for 35 cycles of 30 seconds at 94°C, 50 seconds at 55°C, and 50 seconds at 72°C, followed by a final step of 7 minutes at 72°C. PCR products were detected by agarose gel electrophoresis in 2% NuSieve agarose gels (FMC, USA) and visualized by ethidium bromide staining. The intensity of the bands was quantified using ImageJ software, and the ratios of each gene product normalized to *β*-actin product were considered as the expression of each gene. 

### 2.6. Western Blot Analysis

For western blot analysis, proper size of tissues were completely homogenized and centrifuged at 14000 ×g for 15 minutes at 4°C. The supernatant was collected. An equal volume of 6× SDS sample buffer was added, and the samples were then boiled for 5 minutes. Samples (50 *μ*g per lane) were subjected to electrophoresis on 10% SDS-polyacrylamide gels for 45 minutes at 80 V followed by 100 minutes at 100 V and then transferred onto nitrocellulose for 1 hour at 100 V. The membrane was blocked with 5% skimmed milk for 2 hours at room temperature, then incubated for 1 hour in the presence of anti-HMGB1 monoclonal antibody (diluted 1 : 800, epitomics, Inc., Burlingame, CA, USA), anti-TLR4 polyclonal antibody (diluted 1 : 200, Santa Cruz Biotechnology, Santa Cruz, CA, USA) and *β*-actin (1 : 1000 dilution, Santa Cruz Biotechnology, Santa Cruz, CA, USA) was used as an internal control. After the membrane was washed for 10 minutes each for six times in PBS+Tween 20 (PBST), it was incubated in the appropriate HRP-conjugated secondary antibody (diluted 1 : 400 in PBST) for 2 hours. The blotted protein bands were visualized by enhanced chemiluminescence (ECL) Western blot detection reagents (Amersham, Arlington Heights, IL, USA) and were exposed to X-ray film. Developed films were digitized using an Epson Perfection 2480 scanner (Seiko Corp, Nagano, Japan). Optical densities were obtained using Glyko Bandscan software (Glyko, Novato, CA, USA). 

### 2.7. Nuclear Protein Extract and EMSA

Nuclear protein of the brain tissue was extracted and quantified as previously described [29]. Protein concentration was determined using a bicinchoninic acid assay kit with boving serum albumin as the standard (Pierce Biochemicals, Rockford, Ill, USA). EMSA was performed using a commercial kit (Gel Shift Assay System; Promega, Madison, WI) as previously described [30]. Consensus oligonucleotide probe (5′-AGT TGA GGG GAC TTT CCCAGG C-3′) was end-labeled with [*γ*-^32^P]-ATP (Free Biotech., Beijing, China) with T4-polynucleotide kinase. Nuclear protein (20 *μ*g) was preincubated in a total volume of 20 *μ*L in a binding buffer, consisting of 10 mmol/L Tris-HCl (PH 7.5), 1 mmol/L MgCl_2_, 0.5 mmol/L NaCl, 4% glycerol, 0.5 mmol/L EDTA, 0.5 mmol/L DTT, and 2 *μ*g poly dI-dC for 20 minutes at room temperature. After addition of the 1 *μ*L ^32^P-labled oligonucleotide probe, the incubation was continued for 20 minutes at room temperature. After adding 1 *μ*L of gel loading buffer, the reaction was stopped and the mixture was resolved by electrophoresis on 4% nondenaturing polyacrylamide gel in 0.5× TBE buffer (Tris-borate-EDTA) at 390 V for 1 hour at 4°C. The gel was dried and exposed to X-ray film (Fuji Hyperfilm, Tokyo, Japan) at −70°C with an intensifying screed. Levels of NF-*κ*B DNA binding activity were quantified by computer-assisted densitometric analysis. 

### 2.8. Enzyme-Linked Immunosorbent Assay (ELISA)

The cortex tissue was homogenized with a glass homogenizer in 1 mL of buffer containing 1 mM phenylmethylsulfonyl fluoride, 1 mg/L pepstatin A, 1 mg/L aprotinin, and 1 mg/L leupeptin in phosphate-buffered saline solution (pH 7.2), and then centrifuged at 12,000 ×g for 20 min at 4°C. The supernatant was then collected, and total protein was determined by using a bicinchoninic acid assay kit (Pierce Biochemicals, Rockford, IL, USA). The levels of inflammatory cytokines of the brain tissue were quantified using ELISA kits specific for rat according to the manufacturers' instructions (TNF-*α*, from Diaclone Research, France; IL-1*β*, IL-6 from Biosource Europe SA, Belgium) and previous study of our laboratory [31]. The cytokine contents in the brain tissue were expressed as pg per milligram protein. 

### 2.9. Immunohistochemical Examinations and Cell Counting

Immunohistochemistry was performed to ascertain the immunoreactivity of HMGB1 and TLR4. Briefly, the tissue sections (4 *μ*m) were deparaffinized and rehydrated in gradient alcohol. And then the sections were incubated with anti-HMGB1 monoclonal antibody (diluted 1 : 500, epitomics, Inc., Burlingame, CA, USA) and anti-TLR4 polyclonal antibody (diluted 1 : 100, Santa Cruz Biotechnology, Santa Cruz, CA, USA) overnight at 4°C, followed by a 15-min wash in phosphate-buffered saline. After that the sections were incubated with horseradish peroxidase (HRP-) conjugated IgG (diluted 1 : 500, Santa Cruz Biotechnology Inc., Santa Cruz, CA, USA) for 60 minutes at room temperature. 3, 3′-Diaminobenzidine–H_2_O_2_ solution was used to visualize HMGB1 and TLR4. Those cells that HMGB1 translocated from nucleus to cytoplasm were considered as HMGB1-positive cells. The positive cells were identified, counted, and analyzed in the section through the injured cortex by a pathologist blinded to the experimental group. Five fields within 0.5 mm to the contusion cortex in each section, three sections of each rat brain sample, and five rats in each group were randomly selected and observed under a light microscope (at ×400 magnification) (Eclipse E100, Nikon, Japan). Then, the average percentage of HMGB1 and TLR4 positive cells in each section were calculated as the immunoreactivity of HMGB1 and TLR4 in the rat brains. 

### 2.10. TUNEL Staining and Quantitation of Apoptotic Cells

The formalin-fixed tissues were embedded in paraffin and sectioned at 4 *μ*m thickness with a microtome. The sections were detected for apoptotic cells by terminal deoxynucleotidyl transferase-mediated dUTP nick end labeling (TUNEL) method. An *in situ *cell death detection Kit POD (ISCDD, Boehringer, Mannheim, Germany) was used according to the manufacture's protocol of the kit and our previous study [29]. Microscopic examination of the stained tissue sections was performed by a pathologist blinded to the experimental group. TUNEL-positive cells were counted in the section through the injured cortex. The extent of brain damage was evaluated by apoptotic index, which was the average percentage of TUNEL-positive cells in each section counted in 10 cortical microscopic fields (at ×400 magnification). 

### 2.11. Statistical Analysis

Densitometric quantification and analysis of the PCR, EMSA, and Western blot bands were done with ImageJ software. SPSS 16.0 was used for the statistical analysis (SPSS, Inc., Chicago, IL, USA). All data are expressed as mean ± SEM. The data were subjected to one-way ANOVA followed by Tukey's post-hoc test. Statistical significance was accepted with *P* < .05. 

## 3. Results

### 3.1. Ethyl Pyruvate Improved Beam Walking Balance Performance of Rats after TBI

**Figure 2:**
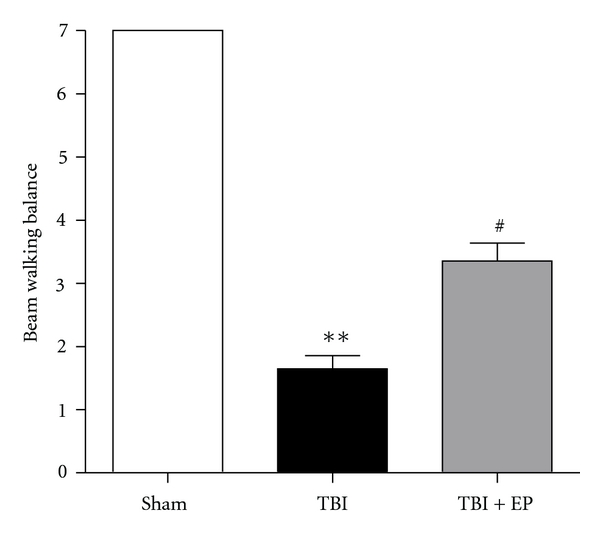
Effects of EP administration on functional outcomes in contusion injured rats as evaluated by beam walking balance tasks. Compared to rats in TBI group, EP administration attenuated the injury induced impairment in beam walking balance performance tested at 24 hours after TBI. Bars represent as mean ± SEM; *n* = 15, each group; **P<.01 versus Sham group, ^#^P<.05 versus TBI group.

All rats had scores of 7 prior to surgical procedure. As shown in Figure 2, the rats in Sham group still had scores of 7 at 24 hours after sham-operation. Compared with Sham group, motor function impairment caused by TBI was significant in the TBI group (*P* < .01). However, EP treatment significantly improved the beam walking balance performance of the rats 24 hours after TBI (*P* < .05) (Figure 2). (*n* = 15, each group; **P<.01 compared with Sham group, ^#^P<.05 compared with TBI group). 

### 3.2. EP Ameliorated Cerebral Edema after TBI

**Figure 3:**
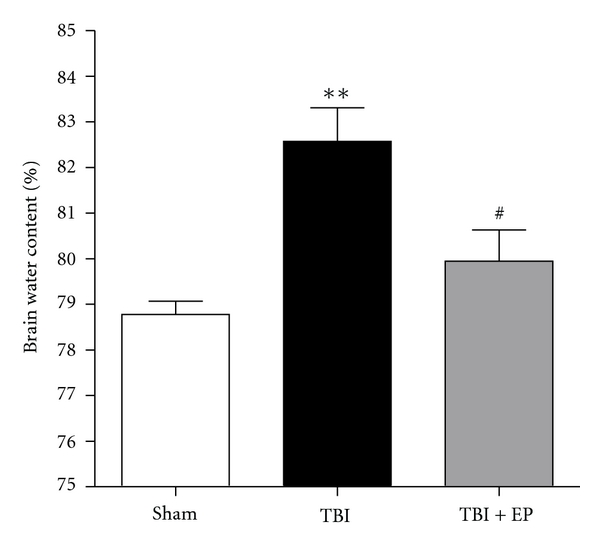
Alteration in brain water content in Sham group, TBI group, and TBI + EP group. The brain water content was increased significantly at 24 hours after TBI. EP administration markedly decreased brain water content 24 h after TBI. Bars represent as mean ± SEM; *n* = 6, each group; **P<.01 versus Sham group, ^#^P<.05 versus TBI group.

Brain water content averaged about 78.9% in the Sham group. Compared to Sham group, significant increase of brain water content was detected in the brain tissue surrounding the injured cortex 24 hours after TBI (*P* < .01). However, the brain water content of injured brain was decreased by EP administration as compared with TBI group (*P* < .01) (Figure 3). These results suggested that treatment with ethyl pyruvate could attenuate rat brain edema following TBI. (*n* = 6, each group; **P<.01 compared with Sham group, ^#^P<.05 compared with TBI group). 

### 3.3. Influence of EP on the mRNA Expression of HMGB1 and TLR4 in the Brain following TBI

**Figure 4:**
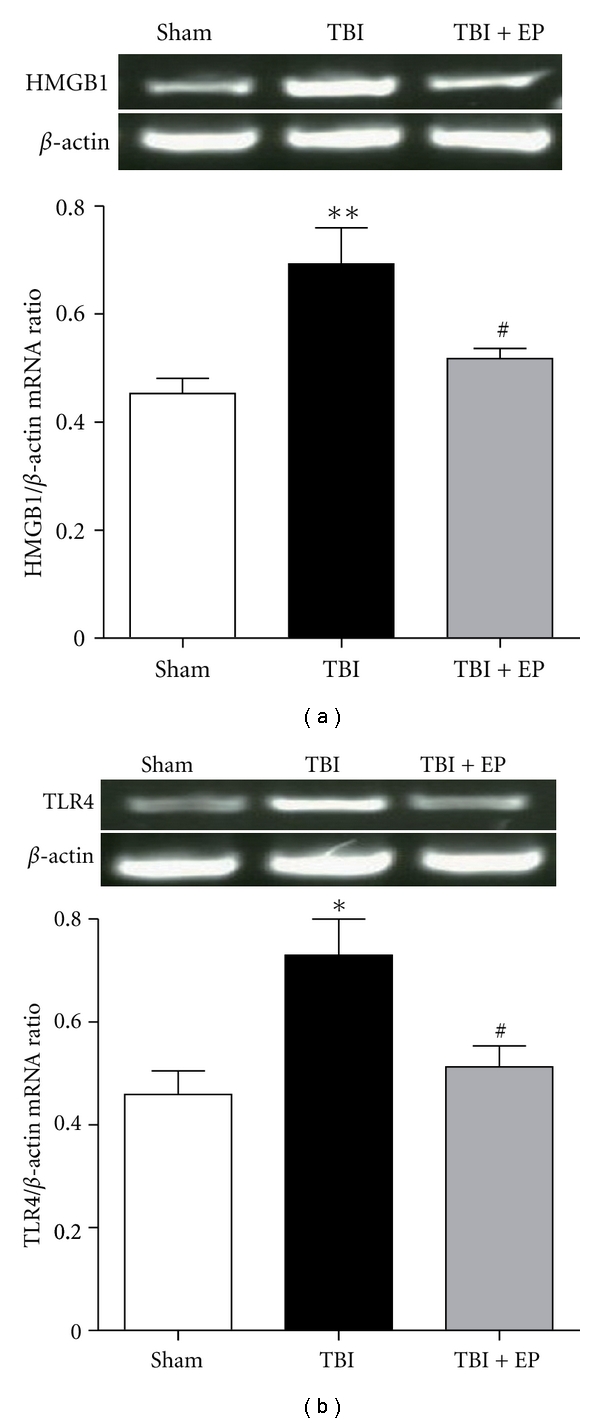
HMGB1 and TLR4 mRNA expression in the rat brain of the Sham group, TBI group, and TBI + EP group. Upper panel: representative RT-PCR bands show the HMGB1 and TLR4 mRNA expression in each group. Bottom panel: density of mRNA bands from each group. Quantitative analysis of the RT-PCR results for the expression of HMGB1 and TLR4 mRNA. It shows that TBI induced a marked increase of HMGB1 and TLR4 mRNA expression in the brain tissue surrounding the contusion cortex compared with the Sham group (*P* < .01). After treatment with EP, the HMGB1 and TLR4 mRNA expressions were significantly downregulated compared to the TBI group (*P* < .05). Bars represent as mean ± SEM; *n* = 6, each group; **P<.01, *P<.05 versus Sham group, ^#^P<.05 versus TBI group.

HMGB1 and TLR4 mRNAs were expressed at low levels in the rat brain of Sham group. The mRNA expression of HMGB1 and TLR4 in the cortex surrounding to the contusion core were significantly increased after TBI compared with that of the Sham group (*P* < .01). But in the EP group, the levels of HMGB1 and TLR4 mRNA in the injured cortex were significantly decreased compared with the TBI group (*P* < .01) (Figure 4). (*n* = 6, each group; *P<.05, **P<.01 compared with Sham group, ^#^P<.05, ^##^P<.01 compared with TBI group). 

### 3.4. Effect of EP on HMGB1 and TLR4 Protein Expression in the Injured Brain

**Figure 5:**
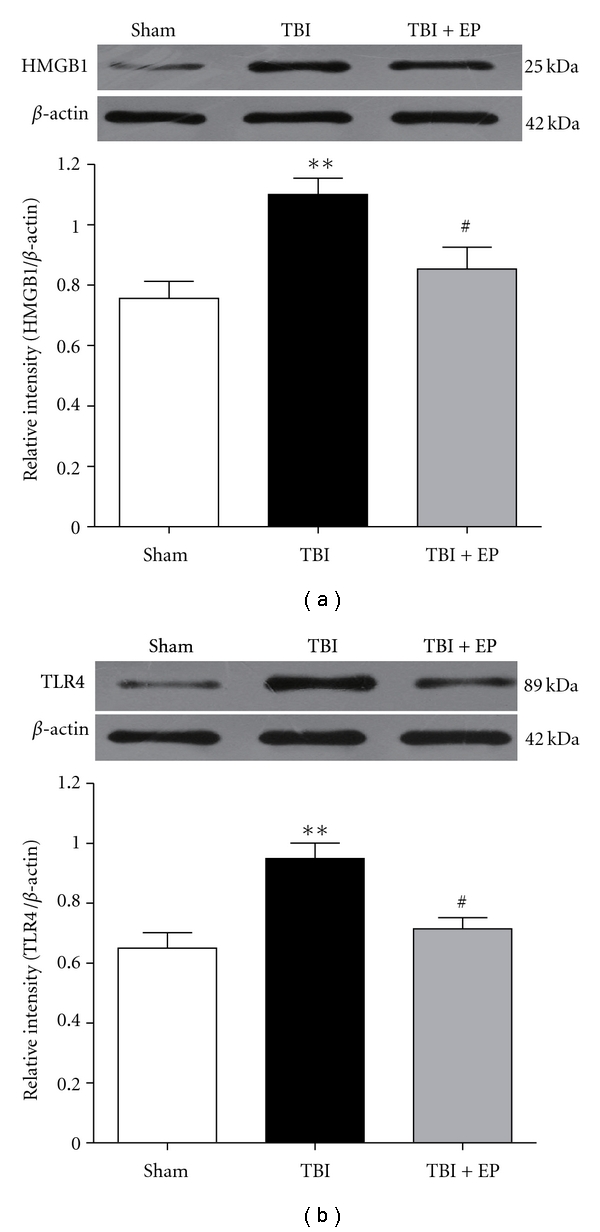
Expression of HMGB1 and TLR4 protein in the rat brain of the Sham group, TBI group, and TBI + EP group. Upper panel represents autoradiogram of the HMGB1 and TLR4 protein expression by Western blot. Bottom panel is quantitative analysis of the Western blot results for the expression of HMGB1 and TLR4 protein. TBI induced a marked increase of HMGB1 and TLR4 protein expression in the brain tissue surrounding the contusion cortex compared to the Sham group (*P* < .01). The HMGB1 and TLR4 protein expressions were significantly downregulated compared to the TBI group after EP treatment (*P* < .05). Bars represent as mean ± SEM; *n* = 6, each group; **P<.01 versus Sham group, ^#^P<.05 versus TBI group.

To determine the influence of EP on HMGB1 and TLR4 expression on injured brain after TBI, Western blot was performed to detect the changes of HMGB1 and TLR4 protein as described in Materials and Methods. The western blot showed low levels of HMGB1 and TLR4 protein in the Sham group (Figure 5). But the protein levels of HMGB1 and TLR4 were significantly increased in the tissue surrounding injured cortex 24 h following TBI compared with Sham group (*P* < .01) (Figure 5). After EP administration, the increased levels of HMGB1 and TLR4 were remarkably suppressed in the TBI + EP group (*P* < .01) (Figure 5). (*n* = 6, each group; *P<.05, **P<.01 compared with Sham group, ^#^P<.05, ^##^P<.01 compared with TBI group). 

### 3.5. EP Administration Inhibited NF-*κ*B DNA Binding Activity after TBI

**Figure 6:**
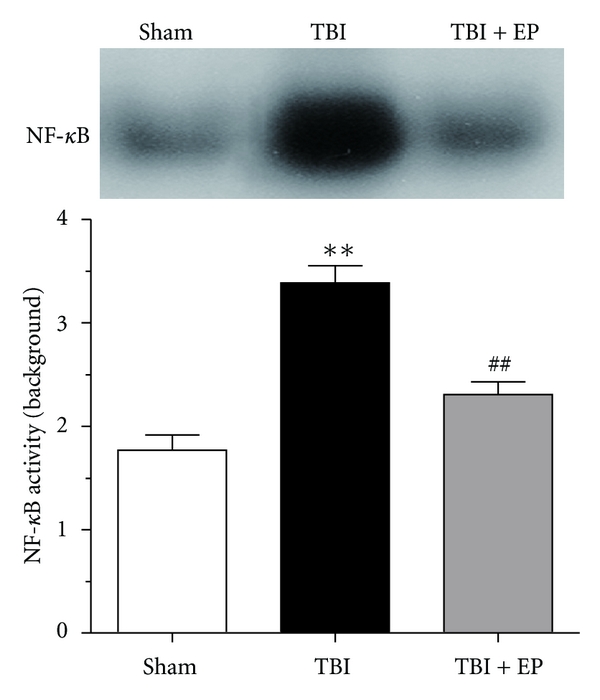
NF-*κ*B DNA binding activity in the brain tissue surrounding the contusion cortex in Sham group, TBI group, and TBI + EP group. Upper panel: EMSA autoradiography of NF-*κ*B DNA binding activity. Lower panel: quantification of NF-*κ*B DNA binding activity was performed by densitometric analysis. As compared with Sham group, NF-*κ*B binding activity measured by EMSA was significantly increased after TBI. However, compared to TBI group, EP treatment significantly suppressed NF-*κ*B binding activation in TBI + EP group. Bars represent as mean ± SEM; *n* = 6, each group; **P<.01 versus Sham group, *P* < .01 versus TBI group.

NF-*κ*B DNA binding activity of the injured brain tissue was assessed by EMSA autoradiography. As shown in Figure 6 upper panel, low NF-*κ*B binding activity (weak EMSA autoradiography) was found in the Sham group. But compared with the Sham group, NF-*κ*B binding activity was significantly increased in the TBI group (*P* < .01). After administering three doses of EP to rats suffered TBI, the NF-*κ*B binding activity was significantly downregulated in the brain tissue surrounding the injured cortex (*P* < .01) (Figure 6 lower panel). (*n* = 6, each group; **P<.01 compared with Sham group, ^##^P<.01 compared with TBI group).

### 3.6. Ethyl Pyruvate Administration Attenuated Production of proinflammatory Cytokines in the Injured Brain after TBI

**Figure 7:**
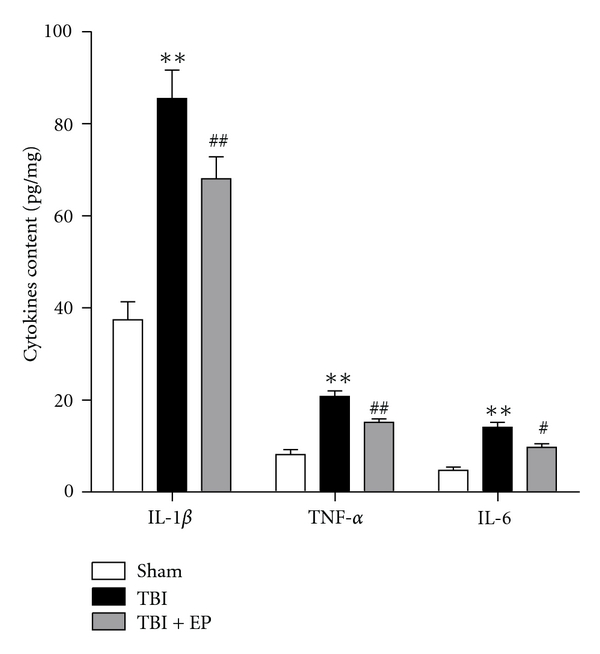
Changes of inflammatory mediators in the injured brain of rats determined by ELISA in Sham group, TBI group, and TBI + EP group. The figure indicated that the levels of TNF-*α*, IL-1*β*, and IL-6 in the injured brain tissue were significantly increased after TBI. In the TBI + EP group, the concentrations of TNF-*α*, IL-1*β*, and IL-6 were markedly downregulated when treated with EP. Bars represent as mean ± SEM; *n* = 6, each group; **P<.01 compared with Sham group, ^#^P<.05, ^##^P<.01 compared with TBI group.

As shown in Figure 7, concentrations of IL-1*β*, TNF-*α* and IL-6 were in low levels in the rat brains of Sham group. But after suffering TBI, the levels of these three cytokines were greatly increased. And EP treatment to the rats after TBI significantly decreased the production of the IL-1*β*, TNF-*α*, and IL-6 in the injured brains. (*n* = 6, each group; *P<.05, **P<.01 compared with Sham group, ^#^P<.05, ^##^P<.01 compared with TBI group). 

### 3.7. Treatment with EP to the Rats after TBI Repressed Percentage of HMGB1 and TLR4 Positive Cells in the Injured Brain

**Figure 8:**
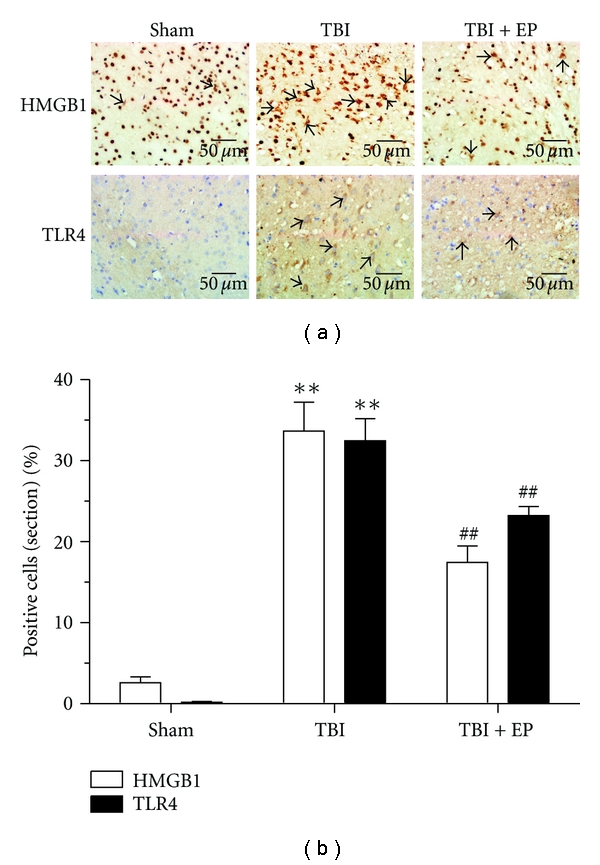
Immunohistochemistry staining showed the expression of HMGB1 and TLR4 protein in the brain tissue. The cytoplasm or extranuclear HMGB1 staining cells were considered as HMGB1-positive cells. (a) Immunohistochemistry staining showed that HMGB1 distributed mostly in the cell nucleus within the Sham group. Few HMGB1 and TLR4 positive cells were observed in the representative cortex. However, in the TBI group, TBI induced HMGB1 redistribution and TLR4 expression. HMGB1 cytoplasm translocation was observed in brain cells. However, EP treatment to the rats significantly decreased HMGB1 translocation and TLR4 expression. Arrows indicated representatively HMGB1 and TLR4 positive cells in the surrounding region of contusion cortex. Scale bars: 50 *μ*m. (b) Quantification of HMGB1 and TLR4 positive cells in each group. TBI induced a marked increase of HMGB1 and TLR4 positive cells in the brain tissue surrounding the contusion cortex compared to the Sham group (*P* < .01). And EP treatment significantly reduced HMGB1 and TLR4 positive cells in TBI + EP group than that compared to the TBI group (*P* < .01). Bars represent as mean ± SEM; *n* = 6, each group; **P<.01 versus Sham group, ^##^P<.01 versus TBI group.

Immunohistochemistry was performed to investigate the localization and expression of HMGB1 and TLR4 in the rat brain. Those brain cells that HMGB1 translocated from nucleus to cytoplasm were considered as HMGB1-positive cells. Few HMGB1 and TLR4-positicve cells were observed in the Sham group. Increased HMGB1 and TLR4-positive cells were present in the TBI group (Figure 8(a)). However, EP treatment to the rats significantly decreased HMGB1 and TLR4 expression. Arrows indicated HMGB1 and TLR4-positive cells in the surrounding region of injured cortex after TBI (Figure 8(a)). Semiquantitative analysis for immunohistochemistry showed the same results as Western blot analysis. In TBI group, the percentage of HMGB1 and TLR4 positive cells were significantly increased as compared with that in the Sham group (*P* < .01). In TBI + EP group, when compared with TBI group, the percentage of HMGB1 and TLR4 positive cells were significantly decreased (*P* < .01) (Figure 8(b)). These results demonstrated that EP could significantly reduce the HMGB1 and TLR4 immunoreactivity in the injured brain after TBI. (*n* = 6, each group; **P<.01 compared with Sham group, ^##^P<.01 compared with TBI group). 

### 3.8. EP Administration Suppressed Cortical Apoptosis in the Injured Brain

**Figure 9:**
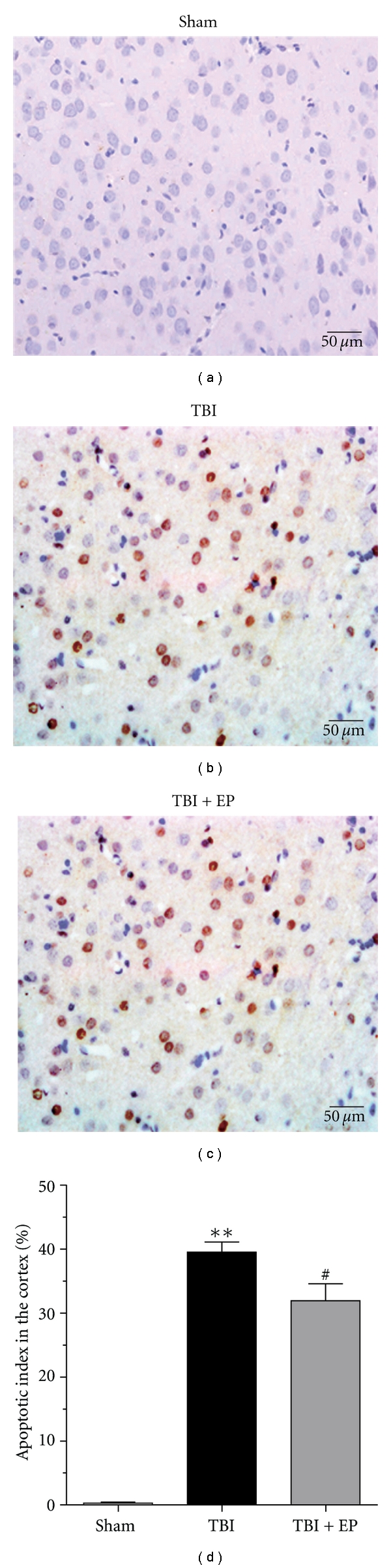
TUNEL immunohistochemistry staining of the injured cortex in Sham group, TBI group, and TBI + EP group. (a) Sham group rats showed few TUNEL apoptotic cells; (b) TBI group rats showed more TUNEL apoptotic cells stained as brown; (c) TBI + EP group rats showed less TUNEL apoptotic cells than TBI group (scale bars, 50 *μ*m). (d) EP administration significantly decreased the apoptotic index in rat injured brain following TBI. Bars represent as mean ± SEM; *n* = 6, each group; **P<.01 versus Sham group, ^#^P<.05 versus TBI group.

Few TUNEL-positive apoptotic cells were found in the rat brains of Sham operated group (Figure 9(a)). In the TBI group, the apoptotic index in the cortex surrounding the injured cortex was significantly increased compared with the Sham group (*P* < .01) (Figures 9(b) and 9(d)). In the TBI + EP group, when compared with that in the TBI group, the apoptotic index in the studied cortex was significantly decreased (*P* < .05) (Figures 9(c) and 9(d)). This result showed that EP treatment after TBI could suppress cell death in the cortex surrounding the injured cortex (Figure 9). (*n* = 6, each group; **P<.01 compared with Sham group, ^#^P<.05 compared with TBI group). 

## 4. Discussion

There is a wealth of evidence to suggest that inflammation plays an important role in the pathophysiology of TBI. TBI initiates the inflammatory response by disrupting the blood-brain barrier, creating edema, and infiltration of inflammatory cells [32]. There is lacking of effective treatment in clinical. Therefore, it is a critical need to develop new pharmacologic therapies for TBI treatment through inhibiting inflammatory responses.

Ethyl pyruvate has been shown to exert cytoprotective actions and anti-inflammatory effects by inhibiting the expression of various proinflammatory mediates as well as HMGB1 in several *in vitro* and *in vivo* models [14, 15]. Recently, EP has been reported to provide a neuroprotective effect on cerebral ischemia [17], spinal cord injury [19], and traumatic brain injury [20].

In the present study, we demonstrated that ethyl pyruvate exerts beneficial effects in a rat weight-dropping model of TBI. The results showed that TBI resulted in brain edema, apoptotic cell death, and motor functional deficits. Meanwhile, traumatic brain injury induced inflammatory responses in the brain tissue, characterized by enhancing NF-*κ*B activation, induced expression of proinflammatory mediates, such as IL-1*β*, TNF-*α*, and IL-6. Also, the expressions of HMGB1 and TLR4 were upregulated remarkably after TBI. Moreover, our results indicated that EP administration reduced (1) brain edema, (2) the mRNA and protein expressions of HMGB1 and TLR4, (3) NF-*κ*B DNA binding activity, (4) proinflammatory cytokines expression, (5) inhibited HMGB1 translocation, (6) suppressed apoptotic cell death, and (7) improved beam walking balance performance in the rats suffered TBI. All of these support the view that EP reduces some degree of secondary inflammation and improves motor function recovery after TBI. To our knowledge, these findings demonstrated for the first time that EP may attenuate the TBI-induced HMGB1/TLR4/ NF-*κ*B signaling pathway activation which may exert on the development of secondary brain damage in rat after TBI.

HMGB1 is a nonhistone nuclear protein with dual function. Inside the cells, HMGB1 binds to DNA and plays a role in transcriptional regulation. Outside the cells, HMGB1 serves as a cytokine-like mediator of inflammation [6]. HMGB1 can be actively or passively released from necrotic or dying cells [7]. Both mechanisms can produce the release of significant amounts of extracellular HMGB1. HMGB1 can bind to cell surface and signal through receptors including RAGE (receptor for advanced glycation end products), TLR2, and TLR4 after being released into the extracellular milieu [6, 11]. Then, these receptors lead to activation of NF-*κ*B signaling and finally promote many cytokines production, such as TNF-*α*, IL-6, and INF-*γ* [6, 33]. In fact, previous studies in our lab demonstrated that TLR2 and TLR4 receptors and NF-*κ*B signaling pathway play a critical role in the inflammatory response to TBI [22, 27, 34]. But the principal TLRs ligands in the brain after TBI remain unknown. Our present study showed that the expression of HMGB1 mRNA and protein were significantly increased (Figures 4(a) and 5(a)) and translocated from nucleus to cytoplasm 24 h after TBI (Figure 8(a)). These results indicate that HMGB1 was involved in the proinflammatory stress response to TBI. HMGB1 may act as one of the TLR4 ligands, signaling through the TLR4/NF-*κ*B pathway which regulated the proinflammatory cytokines production after TBI.

 HMGB1 has been considered as one of the Damage Associate Molecular Patterns (DAMPs) participating in many sterile inflammation diseases, such ischemia reperfusion injury and close trauma [8, 35]. Moreover, HMGB1 was reported upregulated after experimental spinal cord injury and associated with neuronal cell apoptosis [36]. Similar results were obtained from the present study. Treatment with either Anti-HMGB1 antibody or other HMGB1 inhibitors such as EP is beneficial in many preclinical inflammatory disease models [37, 38]. EP was the first described pharmacological inhibitor of HMGB1 secretion. Furthermore, in this study, we showed that EP treatment significantly inhibited HMGB1 expression and cytoplasm translocation (Figures 4(a), 5(a), and 8). Meanwhile, as the same results as previous studies [22, 27], we showed that TLR4, NF-*κ*B binding activation, and proinflammatory mediates, such as IL-1*β*, TNF-*α*, and IL-6 were significantly upregulated after TBI (Figures 4–8). EP treatment to the injured rats could profoundly inhibit the expression of TLR4 and NF-*κ*B DNA binding activation, as well as the expression of several proinflammatory proteins, such as IL-1*β*, TNF-*α*, IL-6, and inflammatory mediates. Recently, Moro and Sutton [20] reported that EP or pyruvate treatments significantly improved recovery of beam walking, neurological scores, and hippocampus neuronal loss after cortical contusion injury. They further indicated that the significant neuroprotection of EP was accompanied by the suppression of microglia activation and proinflammatory cytokines and improved metabolism. Our results further indicated that EP administration following TBI could reduce apoptotic cell death in the injured cortex (Figure 9) and brain edema (Figure 3) and improve motor function recovery (Figure 2). Together, these results indicate that inhibition of HMGB1/TLR4/NF-*κ*B pathway activation by EP resulted in less apoptosis and better functional recovery.

Our study has some limitations. First, we began to treat EP at 5 min after TBI. It is unlikely that EP treatment could begin at this time point in clinical practice, which may limit the clinical use of EP. However, previous research shows that EP suppresses cerebral ischemic injury with a wide therapeutic window as late as 24 hours after stroke [17]. In our further study, we will explore more practical and effective therapeutic window of EP in rat TBI model. Second, we used single EP concentration at 75 mg/kg in this study. In a future study, multiple EP concentrations should be evaluated to confirm the most appropriate EP concentration for maximal neuroprotection in rats after TBI. Third, previous study showed that sodium pyruvate (SP) and EP both have neuroprotective effects in rat model of cortical contusion injury [20]. But, we did not study the protective effects of SP in our present study. We will further confirm whether SP has the same protective mechanisms as EP and the beneficial effects of SP by comparing with EP in TBI model.

In summary, our data demonstrated the effects of EP on the HMGB1/TLR4/NF-*κ*B pathway in the injured brain after TBI. We found that TBI could induce the expressions of HMGB1 and TLR4, downstream factors (IL-1*β*, TNF-*α* and IL-6), and increased NF-*κ*B DNA binding activity in the injured brain, which could be remarkably inhibited by EP treatment. These results suggest that TBI induces activation of HMGB1/TLR4/NF-*κ*B signaling pathway in the injured rat brain which might play a central role in the inflammatory response that may lead to secondary damage after TBI. Inhibition of HMGB1 secretion or release indicates a novel and promising strategy for the treatment of TBI. The neuroprotective effects of EP treatment in weight-dropping TBI rat model may be associated with the inhibition of HMGB1/TLR4/NF-*κ*B pathway activation. Further study on the effects of EP in TBI might provide a novel therapeutic agent for TBI treatment.
