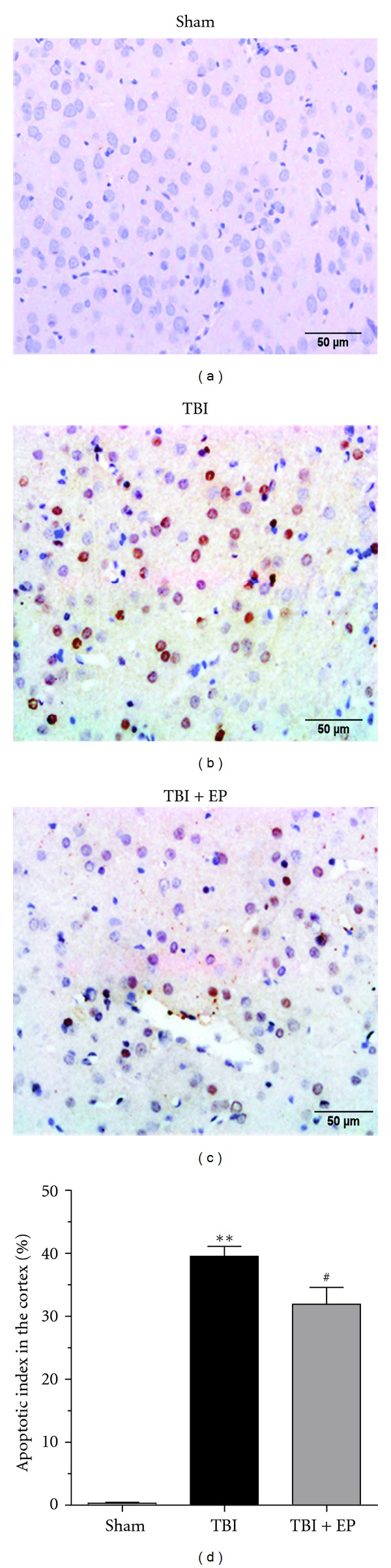# Erratum to “Beneficial Effects of Ethyl Pyruvate through Inhibiting High Mobility Group Box 1 Expression and TLR4/NF-**κ**B Pathway after Traumatic Brain Injury in the Rat”

**DOI:** 10.1155/2012/794531

**Published:** 2012-08-05

**Authors:** Xingfen Su, Handong Wang, Jinbing Zhao, Hao Pan, Lei Mao

**Affiliations:** Department of Neurosurgery, Jinling Hospital, School of Medicine, Nanjing University, 305 East Zhongshan Road, Jiangsu Province, Nanjing 210002, China

There is an error on Figure 9(c) in the original paper. The exact Figure 9 in the original paper has to be corrected as Figure 1 in this paper.

## Figures and Tables

**Figure 1:**